# Biogeography of *Coptis* Salisb. (Ranunculales, Ranunculaceae, Coptidoideae), an Eastern Asian and North American genus

**DOI:** 10.1186/s12862-018-1195-0

**Published:** 2018-05-24

**Authors:** Kun-Li Xiang, Andrey S. Erst, Xiao-Guo Xiang, Florian Jabbour, Wei Wang

**Affiliations:** 1 State Key Laboratory of Systematic and Evolutionary Botany, Institute of Botany, Chinese Academy of Sciences, Beijing, 100093 China; 2 University of Chinese Academy of Sciences, Beijing, 100049 China; 3 Central Siberian Botanical Garden of the Siberian Branch of Russian Academy of Sciences, Zolotodolinskaya str. 101, Novosibirsk, 630090 Russia; 4 Laboratory of Systematics and Phylogeny of Plants, Tomsk State University, Tomsk, 634050 Russia; 5 Institut Systématique, Evolution, Biodiversité (ISYEB), Muséum national d’Histoire naturelle, CNRS, Sorbonne Université, EPHE, 57 rue Cuvier, CP39, 75005 Paris, France

**Keywords:** Ancestral range evolution, Climate change, *Coptis*, Eastern Asian, Taiwan, Western North America

## Abstract

**Background:**

Numerous studies have favored dispersal (colonization) over vicariance (past fragmentation) events to explain eastern Asian-North American distribution patterns. In plants, however the disjunction between eastern Asia and western North America has been rarely examined using the integration of phylogenetic, molecular dating, and biogeographical methods. Meanwhile, the biogeographic patterns within eastern Asia remain poorly understood. The goldthread genus *Coptis* Salisb. includes 15 species disjunctly distributed in North America, Japan, mainland China, and Taiwan. We present a dated phylogeny for *Coptis* under the optimal clock model and infer its historical biogeography by comparing different biogeographic models.

**Results:**

The split of *Coptis* and *Xanthorhiza* Marshall occurred in the middle Miocene (ca. 15.47 Ma). *Coptis* started their diversification in the early late Miocene (ca. 9.55 Ma). A late Miocene vicariance event resulted in the eastern Asian and western North American disjunction in the genus. Within eastern Asia, dispersals from mainland Asia to Japan and from Japan to Taiwan occurred at ca. 4.85 Ma and at ca. 1.34 Ma, respectively.

**Conclusions:**

Our analyses provide evidence that both vicariance and dispersal events have played important roles in shaping the current distribution and endemism of *Coptis*, likely resulting from eustatic sea-level changes, mountain formation processes and an increasing drier and cooler climate from the middle Miocene onwards.

**Electronic supplementary material:**

The online version of this article (10.1186/s12862-018-1195-0) contains supplementary material, which is available to authorized users.

## Background

Understanding the geographical deployment of biodiversity through time is a central theme in historical biogeography [1]. The disjunct distributions of closely related organisms between East Asia and North America have fascinated botanists and biogeographers for over a century and a half [2–5]. In plants, biogeographic studies employing the integration of phylogenetic hypotheses, inference of ancestral ranges, and estimates of divergence times have largely focused on the classic eastern Asian and eastern North American floristic disjunction pattern [5–8]. Few studies have been devoted to investigate the eastern Asian and western North American disjunction [9, 10]. For these two patterns, the Miocene has been regarded as an important period, in which the Bering land bridge likely acted as a major gateway [5, 11–13].

**Fig. 1:**
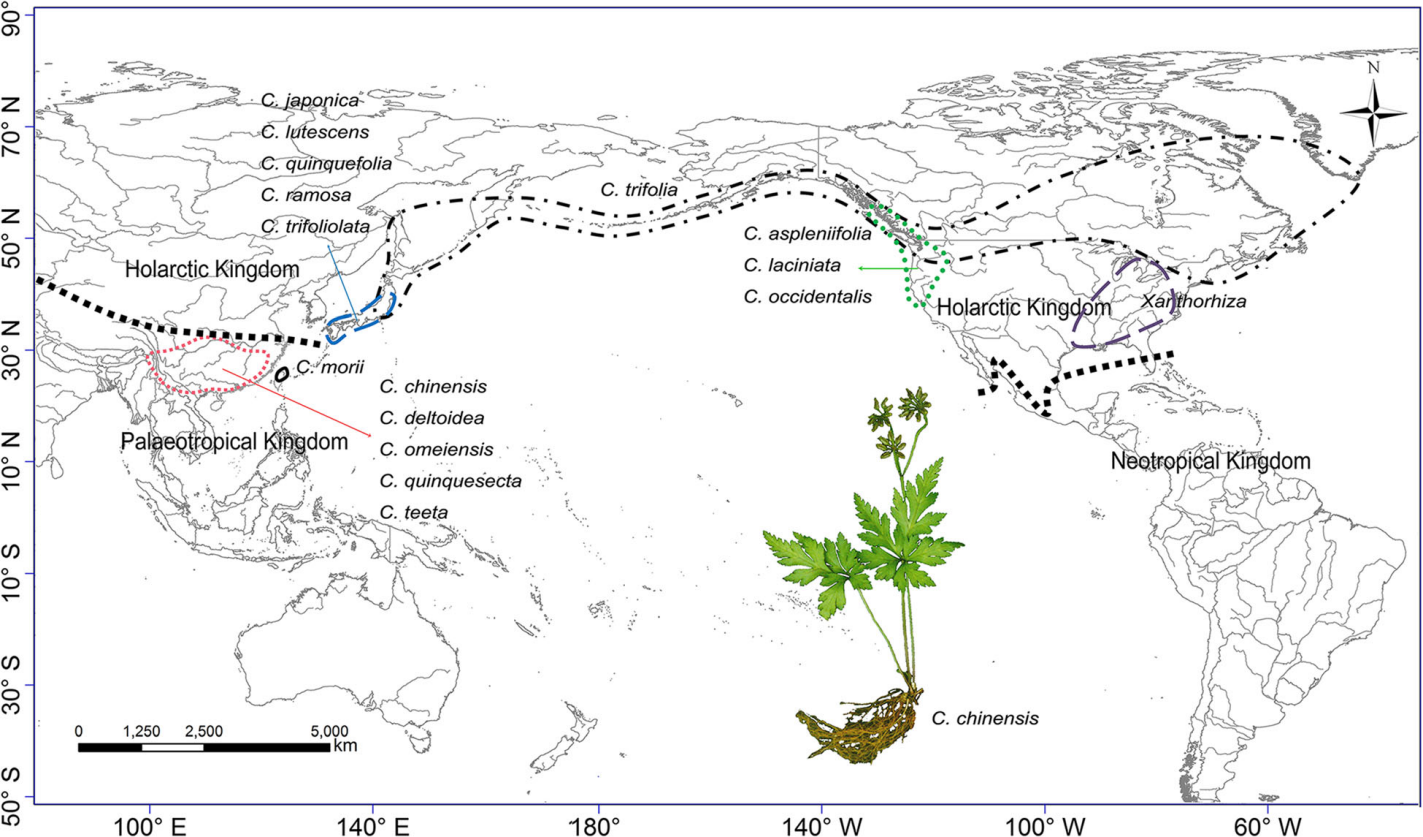
Geographic range of *Coptis* species. Doted lines in bold demarcate boundaries of the Holarctic and Paleotropical kingdoms according to Takhtajan [16]

In the Northern Hemisphere, East Asia is a pivotal biogeographic region as it presents high levels of plant species diversity and endemism [14, 15]. Based on Takhtajan’s [16] floristic system, southern East Asia belongs to the Paleotropical Kingdom, whereas northern East Asia is part of the Holarctic Kingdom (Fig. 1). Recent molecular phylogenetic studies also indicate that the Tertiary relict floras within East Asia could be subdivided into two distinct southern and northern regions [17, 18]. The former consists of southern and southeastern China with extending to the Himalayas, while the latter contains Japan, Korea, and northeastern China. Besides, as a continental island adjacent to southeastern mainland China, the Ryukyus Islands, and Philippines, the floristic source of Taiwan is not clear [19–21]. To date, biogeographic relationships among southern East Asia, northern East Asia and Taiwan are far from understood.

The goldthread genus *Coptis* Salisb. (Ranunculales, Ranunculaceae, Coptidoideae) is of pharmaceutical and economical importance and is mainly distributed in the warm temperate to the cold coniferous forests of eastern Asia and North America [22, 23]. Among the 15 species recognized by Tamura [22], *C. trifolia* (L.) Salisb. has the widest distribution area (including Japan, the Kurile Islands, Kamchatka, and North America), while the other 14 species are restricted to smaller regions: five species are found in southern and southwestern mainland China with extensions to the Himalayas, five in Japan, one in Taiwan, and three in western North America (Fig. 1). Our recent phylogenetic analysis based on three DNA markers indicates that three western North American species of the genus clustered with five mainland Chinese and two Japanese species, and Taiwanese *C. morii* Hayata and three Japanese species grouped together [23]. The fruits of *Coptis* are dehiscent follicles [22] and seeds may be autochorously dispersed owing to lacking obvious adaptation to wind-dispersal. Seeds are not thereby expected to disperse over long distance or oceanic barriers. Thus, *Coptis* provides a remarkable opportunity for studying the eastern Asian and western North American distribution pattern, as well as the biogeographic relationships within East Asia.

In this study, first we reconstruct a dated phylogeny for *Coptis* based on six DNA markers, using a Bayesian relaxed clock method. Using the resulting dated-phylogenetic framework, we then infer the ancestral range evolution of *Coptis* by comparing the relative fit of six biogeographic models. Our study contributes to the knowledge on the eastern Asian-western North American distribution pattern and eastern Asian biogeography.

## Methods

### Samples and sequences

We sampled all 15 species of *Coptis* recognized by Tamura [22]. *Coptis* and the monotypic *Xanthorhiza* Marshall compose the subfamily Coptidoideae, which is sister to a large clade containing the overwhelming majority of genera of Ranunculaceae [24, 25]. Scoring this large clade for geographic areas is a challenge. Here, we only selected *Xanthorhiza* as the outgroup. The sampled species and their GenBank accession numbers are listed in Additional file 1: Table S1.

Six DNA markers, including five plastid (*rbcL*, *trnL* intron, *trnL-F* spacers, *trnD-trnT*, and *trnH-psbA*) and one nuclear (ITS) regions were used in this study. We generated new *trnL* sequence for *C. japonica* var. *anemonifolia* (Siebold & Zucc.) H. Ohba and *trnL* and ITS for *C. morii*. These two samples were collected in public land and no specific permits were required. Other sequences were obtained from GenBank. Laboratory procedures and sequence handling followed Wang and Chen [26]. Three difficult-to-align regions in *trnL-F* (encompassing 20 positions), two difficult-to-align regions in *trnH-psbA* (48 positions), and one difficult-to-align region in *trnD-trnT* (24 positions) were excluded from the analyses. The final dataset included 4288 characters: *rbcL*, 1304 bp; *trnL* intron, 465 bp; *trnL-F*, 426 bp; *trnD-trnT*, 1122 bp; *trnH-psbA*, 289 bp; and ITS, 682 bp.

### Phylogeny and divergence time estimates

We first conducted a likelihood ratio test [27] to determine whether our sequence data were evolving in a clock-like fashion. Because rate constancy along all branches of the phylogeny was rejected (δ = 146.63, d.f. = 14, *P* < 0.0001), we used a Bayesian relaxed clock methodology as implemented in BEAST v1.8.2 [28] to generate a dated phylogeny for *Coptis*. Based on our recent broader study of Ranunculaceae [25], the split time between *Coptis* and *Xanthorhiza* was estimated at ca. 16.23 Ma (95% highest posterior density (HPD): 8.51–25.96) and was here used as a secondary calibration point. Following the suggestion of Ho [29], we assigned a prior normal distribution for the calibration, in which a standard deviation of 2 was set.

Following the result of Baele et al. [30], we used Bayes factors [31] calculated by marginal likelihoods derived from path sampling (PS) [32] and stepping-stone sampling (SS) [33] to compare the parametric fit of three clock models: exponential, lognormal and random. Since our sampling included all recognized species of *Coptis* and *Xanthorhiza*, a birth-death tree prior was used.

For all BEAST analyses, data partitioning and nucleotide substitution models were determined using PartitionFinder 2.1.1 [34, 35]. The Markov chain Monte Carlo chains were run for 100 million generations, sampling every 10,000 generations. Tracer v1.6 [36] was used to assess appropriate burn-in and the adequate effective sample size values (> 200). A burn-in of 25% was applied, and the maximum clade credibility (MCC) tree with the mean ages and 95% HPD intervals on nodes were conducted in TreeAnnotator v1.8.2 (part of the BEAST package) and edited in FigTree v.1.4.2 (http://beast.bio. ed.ac.uk/FigTree).

### Ancestral range analysis

Based on the floristic characteristics [16, 18] and distributions of *Coptis* and *Xanthorhiza* [22], we coded five biogeographical areas (Fig. 1): (A) western North America, (B) southern East Asia (including southern and southeastern mainland China and the adjacent Himalayan region), (C) Japan and adjacent islands (including the Kurile Islands and Kamchatka), (D) Taiwan, and (E) eastern North America. The maximum range size was set to three, as no extant species occurs in more than three biogeographical regions. Because the Bering land bridge was periodically available for exchanges of plants between eastern Asia and western North America until 3.5 Ma [37–39], dispersal probabilities between pairs of areas were specified for two separate time slices (Additional file 1: Table S2).

We used the R package BioGeoBEARS [40] for ancestral range estimation (ARE) on the MCC tree from the BEAST run under the optimal clock model and tree speciation prior. Recently, Ree & Sanmartín [41] demonstrated that the likelihood-based models with the +J parameter are invalid because of errors in the estimation of likelihoods. Here we compared the following three models of biogeographical estimation in the maximum likelihood (ML) framework: dispersal-extinction cladogenesis (DEC) model [42], dispersal–vicariance analysis (DIVA) [43] and BayArea model [44]. The fit for the different models was assessed using the Akaike information criterion scores.

## Results

### Phylogeny and divergence times

**Fig. 2:**
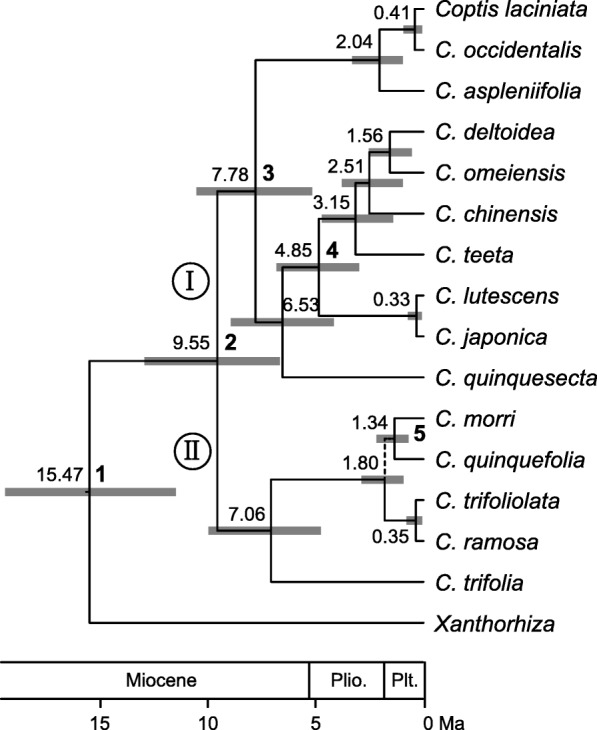
Dated phylogeny of *Coptis* inferred from the combined plastid and nuclear data using BEAST under the random clock model and birth-death tree prior. Gray bars represent 95% highest posterior density intervals. Nodes of interests were marked as 1–5 in bold. All nodes are strongly supported (PP > 0.95) except for one node (in dashed line). Plio., Pliocene; Plt., Pleistocene

We identified the random clock model as optimal for our data (Table 1). The dated phylogenetic tree generated in the BEAST analysis under the random clock model and birth-death tree prior is indicated in Fig. 2. The relationships among *Coptis* species are well resolved with strong support (PP > 0.95) except for the node defining the sister relationship of *C. quinquefolia* Miq. and *C. morii*. *Coptis* contains two main clades (I and II). Based on our time estimates (Fig. 2), the stem and crown ages of *Coptis* are estimated at ca. 15.47 Ma (95% HPD: 11.47–19.37; node 1) and 9.55 Ma (95% HPD: 6.66–12.92; node 2), respectively. Within clade I, three western North American species clustered together and split from their eastern Asian sister group at ca. 7.78 Ma (95% HPD: 5.16–10.52; node 3). Japanese *C. japonica* Makino and *C. lutescens* Tamura are nested in the group of mainland Chinese species and the split of these two Japanese species and their sister group occurred at ca. 4.85 Ma (95% HPD: 2.98–6.80; node 4). Within clade II, Taiwanese *C. morii* and Japanese *C. quinquefolia* were grouped together with weak support (PP = 0.73). The split time of *C. morii* and *C. quinquefolia* was estimated to be at *ca.* 1.34 Ma (95% HPD: 0.69–2.18; node 5).

**Table 1:** Comparison of three clock models in BEAST analyses via Bayes factors

Clock model	Marginal likelihood	Exponential	Lognormal	Random
PS implementation				
Exponential	− 8809.70	–	37.44	−32.90
Lognormal	− 8828.42	−37.44	–	−70.34
Random	− 8793.25	32.90	70.34	
SS implementation				
Exponential	− 8810.29	–	37.62	−33.72
Lognormal	− 8829.10	−37.62	–	−71.34
Random	−8793.43	33.72	71.34	–

2ln Bayes factor (BF) was calculated by marginal likelihoods derived from path sampling (PS) and stepping-stone sampling (SS) implementations in BEAST. 2ln BF > 2.0 represents positive evidence, > 6.00 represents strong evidence, and > 10.00 represents very strong evidence [31]

### Ancestral range estimation

**Fig. 3:**
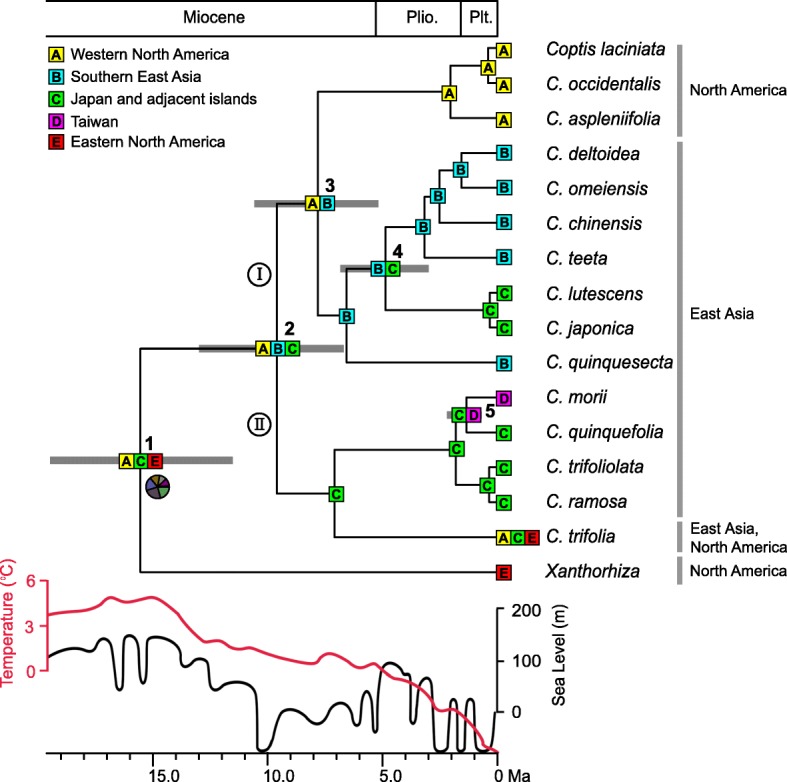
Ancestral range estimation (ARE) for *Copits* BEAST using BioGeoBEARS under the DIVALIKE model. Labeled nodes (1 to 5, as referred to Fig. 2), with 95% highest posterior densities (grey bars), are discussed in the text. The estimated ancestral ranges with the highest ML probability are shown by boxes on each node. Additional file 2: Figure S1 provides all ARE per node with pies. A pie is placed in this figure at the root with the highest probability less than 50%. The depictions of temperature (in red) and sea level (in black) changes are modified from Zachos et al. [45] and Haq et al. [57], respectively. Plio., Pliocene; Plt., Pleistocene

A DIVALIKE was found to be the best-fitting model (Table 2). The ARE for *Coptis* using BioGeoBEARS is indicated in Fig. 3 and Additional file 2: Figure S1. Area probabilities of all nodes are high except the root. Our ARE shows that the ancestral range of *Coptis* and *Xanthorhiza* is unresolved but likely involved eastern North America, western North America and Japan (node 1). The most recent common ancestor of *Coptis* was likely distributed in western North America, southern East Asia and Japan (node 2). Within *Coptis*, two vicariance events and two dispersal events were inferred at the species level (Fig. 3).

**Table 2:** Comparison of the fit of three models of biogeographical range evolution and model-specific estimates for the different parameters

Model	Ln*L*	Parameter nb	*d*	*e*	AIC	ΔAIC	AIC_C_	ΔAIC_C_
DEC	−24.06	2	0.03	1.00 × 10^− 12^	52.12	3.59	53.04	3.58
DIVALIKE	−22.27	2	0.03	1.00 × 10^− 12^	48.53	0	49.46	0
BAYAREALIKE	−28.77	2	0.04	1.04 × 10^−1^	61.55	13.02	52.47	13.01

*d* = dispersal rate; *e* = extinction rate

## Discussion

The phylogenetic relationships in *Coptis* are highly consistent with the results of Xiang et al. [23], but are usually resolved with greater support for clades found therein. Our results do not support Taiwanese *C. morii* as sister to three Japanese species (*C. ramose* (Makino) Tamura, *C. quinquefolia* and *C. trifoliolata* (Makino) Makino), and instead suggest that *C. morii* is sister to *C. quinquefolia*, although with moderate support (PP = 0.71). Using the split age of ca. 16.23 Ma (95% HPD: 8.51–25.96) between *Coptis* and *Xanthorhiza* [25], we obtained a similar age estimate for the split (ca. 15.47 Ma; 95% HPD: 11.47–19.37; Fig. 2).

BioGeoBEARS analyses indicate that the crown of *Coptis* and *Xanthorhiza* most likely occurred in a widespread area comprising North America and Japan (Fig. 3; node 1), although other somewhat less likely ARE are possible (Additional file 2: Figure S1). The estimated age for the split of these two genera highly coincides with the mid-Miocene Climatic Optimum (MMCO; ~ 15–17 Ma; Fig. 3) [45]. During this period, exchange of temperate plants between East Asia and North America could occur via the Bering land bridge [46]. Paleobotanical data indicate that the mixed mesophytic forest of the early and middle Miocene was continuous from Japan through Alaska and into conterminous North America [47, 48].

The American west encompassing the Colorado Plateau, Basin and Range, the High Plains, and the Rocky and Sierra Mountains began to uplift rapidly by 20–15 Ma [49]. A middle Miocene flora from Carson Pass in the central Sierra Nevada suggests uplift of about 2300 m since that time [50]. The uplift is a key factor in creating an increasingly drier climate in the North American interior around that time [49, 51]. Paleobotanical evidence suggests that by the middle Miocene the arid interior has become an effective barrier to biotic interchange between eastern and western North America [52, 53]. After the MMCO, an increasingly drier climate, as well as global cooling (Fig. 3) [45], might thus have resulted in a vicariance event responsible for the divergence of *Coptis* and *Xanthorhiza* (node 1; Fig. 3).

After *Coptis* diverged from *Xanthorhiza*, a subsequent dispersal from Japan to southern East Asia occurred in the early late Miocene (9.55 Ma, 95% HPD: 6.66–12.92; node 2). This time is markedly later than the time of the opening of the Japan Sea (23–15 Ma), which separated the Japanese Islands from the Northeast Asian margins [54–56]. However, during the early late Miocene, a marked drop of sea level occurred (Fig. 3) [57], which might have resulted in East China Sea seafloor exposure between the Eurasian mainland and the Japanese Archipelago. Hence, *Coptis* could have migrated westward into continental Asia via this land bridge. Subsequent sea-level rise might have resulted in the interruption of population exchange of the genus between the Asian mainland and the Japanese Islands. Accordingly, *Coptis* diverged into two clades (I and II).

In clade I, one vicariance episode happened between western North America and southern East Asia in the Late Miocene (ca. 7.78 Ma, 95% HPD: 5.16–10.52; node 3), which overlapped closely with the time of the first opening of the Bering Strait (7.4–5.5 Ma) [58]. Evidence from sedimentology and foraminifera indicates that uplift of the St. Elias Mts. in Alaska began about 8.5 Ma [59]. Palynological analyses suggest that the trends of temperature decline and increasing canopy openness in Alaska and Yukon Territory occurred between 9.7 and 7.0 Ma, owing to global and local tectonic developments [60]. These events may explain the distribution of *Coptis* between southern East Asia and western North America during the Late Miocene. The split of western North American *Polypodium californicum* Kaulf. (Polypodiaceae) and its eastern Asian relatives (*P. fauriei* (Copel.) Makino & Nemoto and *P. glycyrrhiza* D.C. Eaton) also occurred during the same period (ca. 8.81 Ma, 95% HPD: 5.06-13.08) [61]. Such distribution patterns resulting from orogenic events have been found in some plant lineages and in different biomes, such as Campanulaceae [62], Orchidaceae [63], and Rubiaceae [64].

One dispersal event in clade I occurred in the early Pliocene from southern East Asia to Japan (ca. 4.85 Ma, 95% HPD: 2.98–6.80; node 4). The most recent common ancestor of Japanese *Pseudotsuga japonica* (Shiras) Beissn. and mainland Chinese *P. gaussenii* Flous and *P. sinensis* Dode (Pinaceae) was estimated to occur at ca. 4.64 ± 1.93 Ma [65]. In Eupteleaceae, Chinese *Euptelea pleiosperma* Hook. f. & Thomson split with Japanese *E. polyandra* Siebold & Zucc. at ca. 6.04 Ma (95% HPD: 2.89–9.36) [66]. The drop of sea level may have resulted in exchanges of plants between mainland Asia and the Japanese Islands via the East China Sea land bridge, and subsequent rise of sea level and global cooling (Fig. 2) [67], as well as an increasingly drier climate in Asia [68], may have caused the interruption of the continuous distribution of ancestral populations of some extant species during the Late Miocene to the Early Pliocene.

Within clade II, one dispersal event from Japan to Taiwan occurred in the Early Pleistocene (ca. 1.34 Ma; 95% HPD: 0.69–2.18; node 5). The eustatic sea-level fluctuation during this period, as well as global cooling (Fig. 2), may have triggered *Coptis* range expansion from Japan to Taiwan via the Ryukyu Islands, and may have subsequently caused range fragmentation*.* A similar scenario also explains the current distribution of Taiwanese *Chamaecyparis formosensis* Matsum. and *C. taiwanensis* Masam. & Suzuki (Cupressaceae) from hypothetical Japanese ancestors [69]. Our analysis on *Dichocarpum* W.T. Wang & P.G. Xiao indicates that Taiwanese *D. arisanense* (Hayata) W.T. Wang & P.G. Xiao could have originated from mainland China in the Early Pleistocene (ca. 1.26 Ma, 95% HPD: 0.48–2.33) [70]. These studies support the hypothesis that temperate elements of the flora of Taiwan recently migrated from mainland China and Japan [71].

## Conclusions

We present a dated phylogeny for all species of *Coptis*, a genus of pharmaceutical and economical importance. Our biogeographical inference indicates that a vicariance event between Japan-western North America and eastern North America occurred in the Middle Miocene, resulting in the split of *Coptis* and *Xanthorhiza*. The most recent common ancestor of *Coptis* occurred in western North America, southern East Asia and Japan. In *Coptis*, two vicariance episodes, involving Japan and western North America-southern East Asian and western North America and southern East Asian, took place at ca. 9.55 Ma and 7.78 Ma, respectively. Two dispersal events happened from mainland Asia to Japan at ca. 4.85 Ma and from Japan to Taiwan at ca. 1.34 Ma, respectively. This study shed light on the past floristic exchanges between East Asia and North America, as well as within East Asia.

## Additional files

Additional file 1: **Table S1.** GenBank accession numbers and vouchers/references for the sequences used in this study. **Table S2.** Manual dispersal multipliers. (PDF 36 kb)

Additional file 2: **Figure S1.** Raw PDF outputs from biogeographic estimations in BioGeoBEARS. (PDF 358 kb)

## Abbreviations

ARE: Ancestral range estimation
BF: Bayes factor
DEC: Dispersal-extinction cladogenesis
DIVA: Dispersal–vicariance analysis
HPD: Highest posterior density
MCC: Maximum clade credibility
ML: Maximum likelihood
MMCO: Mid-Miocene Climatic Optimum
PS: Path sampling
SS: Stepping-stone sampling

## References

[1] Lomolino MV, Riddle BR, Whittaker RJ, Brown JH (2010). Biogeography.

[2] Sanmartín I, Enghoff H, Ronquist F (2001). Patterns of animal dispersal, vicariance and diversification in the Holarctic. Biol J Linn Soc.

[3] Gray A (1859). Diagnostic characters of phanerogamous plants collected in Japan by Charles Wright, botanist of the U.S. North Pacific exploring expedition, with observations upon the relations of the Japanese flora to that of North America, and other parts of the northern temperate zone. Mem Am Acad Arts Sci NS.

[4] Li HL (1952). Floristic relationships between eastern Asia and eastern North America. Trans Am Phil Soc.

[5] Wen J, Ickert-Bond SM, Nie ZL, Li R, Long M, Gu H, Zhou Z (2010). Timing and modes of evolution of eastern Asian–north American biogeographic disjunctions in seed plants. Darwin’s heritage today: proceedings of the Darwin 200 Beijing international conference.

[6] Donoghue MJ, Bell CD, Li JH (2001). Phylogenetic patterns in northern hemisphere plant geography. Int J Plant Sci.

[7] Donoghue MJ, Smith SA (2004). Patterns in the assembly of the temperate forest around the northern hemisphere. Phil Trans R Soc Lond B.

[8] Deng T, Nie ZL, Drew BT, Volis S, Kim C, Xiang CL, Zhang JW, Wang YH, Sun H (2015). Does the Arcto-tertiary biogeographic hypothesis explain the disjunct distribution of northern hemisphere herbaceous plants? The case of *Meehania* (Lamiaceae). PLoS One.

[9] Zhou Z, Wen J, Li GD, Sun H (2012). Phylogenetic assessment and biogeographic analyses of tribe Peracarpeae (Campanulaceae). Plant Syst Evol.

[10] Wen J, Nie ZL, Ickert-Bond SM (2016). Intercontinental disjunctions between eastern Asia and western North America in vascular plants highlight the biogeographic importance of the Bering land bridge from late cretaceous to Neogene. J Syst Evol.

[11] Wang W, Chen ZD, Liu Y, Li RQ, Li JH (2007). Phylogenetic and biogeographic diversification of Berberidaceae in the northern hemisphere. Syst Bot.

[12] Wen J (1999). Evolution of eastern Asian and eastern north American disjunct distributions in flowering plants. Annu Rev Ecol Syst.

[13] Wang XQ, Ran JH (2014). Evolution and biogeography of gymnosperms. Mol Phylogenet Evol.

[14] Wu ZY, Wu SG, Zhang AL, Wu SG (1996). A proposal for a new floristic kingdom (realm) — the E. Asiatic kingdom, its delimitation and characteristics. Floristic characteristics and diversity of east Asian plants.

[15] Manchester SR, Chen ZD, Lu AM, Uemura K (2009). Eastern Asian endemic seed plant genera and their paleogeographic history throughout the northern hemisphere. J Syst Evol.

[16] Takhtajan A (1986). Floristic regions of the world.

[17] Milne RI, Abbott RJ (2002). The origin and evolution of tertiary relict floras. Adv Bot Res.

[18] Milne RI (2006). Northern hemisphere plant disjunctions: a window on tertiary land bridges and climate change?. Ann Bot.

[19] Fang BZ, Zhuo ZD (1995). Basic features of seed plant flora in Taiwan area. Trop Geogr.

[20] Ying TS, Hsu KS (2002). An analysis of the flora of seed plants of Taiwan, China: its nature, characteristics, and relations with the flora of the mainland. Acta Phytotax Sin.

[21] Hsieh CF (2002). Composition, endemism and phytogeographical affinities of the Taiwan flora. Taiwania.

[22] Tamura M, Hiepko P (1995). Coptis. Die natürlichen Pflanzenfamilien.

[23] Xiang KL, Wu SD, Yu SX, Liu Y, Jabbour F, Erst AS, Zhao L, Wei W, Chen ZD (2016). The first comprehensive phylogeny of Coptis (Ranunculaceae) and its implications for character evolution and classification. PLoS One.

[24] Wang W, Lu AM, Ren Y, Endress ME, Chen ZD (2009). Phylogeny and classification of Ranunculales: evidence from four molecular loci and morphological data. Perspect Plant Ecol Evol Syst.

[25] Wang W, Lin L, Xiang XG, Ortiz RC, Liu Y, Xiang KL, Yu SX, Xing YW, Chen ZD (2016). The rise of angiosperm-dominated herbaceous floras: insights from Ranunculaceae. Sci Rep.

[26] Wang W, Chen ZD (2007). Generic level phylogeny of Thalictroideae (Ranunculaceae) — implications for the taxonomic status of *Paropyrum* and petal evolution. Taxon.

[27] Felsenstein J (1981). Evolutionary trees from DNA sequences: a maximum likelihood approach. J Mol Evol.

[28] Drummond AJ, Suchard MA, Xie D, Rambaut A (2012). Bayesian phylogenetics with BEAUti and the BEAST 1.7. Mol Biol Evol.

[29] Ho SYW (2007). Calibrating molecular estimates of substitution rates and divergence times in birds. J Avian Biol.

[30] Baele G, Li WLS, Drummond AJ, Suchard MA, Lemey P (2013). Accurate model selection of relaxed molecular clocks in Bayesian phylogenetics. Mol Biol Evol.

[31] Kass RE, Raftery AE (1995). Bayes factors. J Amer Stat Assoc.

[32] Lartillot N, Philippe H (2006). Computing Bayes factors using thermodynamic integration. Syst Biol.

[33] Xie W, Lewis PO, Fan Y, Kuo L, Chen MH (2011). Improving marginal likelihood estimation for Bayesian phylogenetic model selection. Syst Biol.

[34] Lanfear R, Calcott B, Ho SYW, Guindon S (2012). PartitionFinder: combined selection of partitioning schemes and substitution models for phylogenetic analyses. Mol Biol Evol.

[35] Lanfear R, Frandsen PB, Wright AM, Senfeld T, Calcott B (2016). PartitionFinder 2: new methods for selecting partitioned models of evolution for molecular and morphological phylogenetic analyses. Mol Biol Evol.

[36] Rambaut A, Suchard MA, Xie D, Drummond AJ. Tracer, version 1.6. 2014. http://beast.bio.ed.ac.uk/Tracer/. Accessed 9 July 2017.

[37] Allen RT (1983). Distribution patterns among arthropods of the north temperate deciduous forest biota. Ann Missouri Bot Gard..

[38] Tiffney BH (1985). Perspectives on the origin of the floristic similarity between eastern Asia and eastern North America. J Arn Arb.

[39] Cunningham CW, Collins TM, Schierwater B, Streit B, Wagner GP, DeSalle R (1994). Developing model systems for molecular biogeography: vicariance and interchange in marine invertebrates. Molecular ecology and evolution, approaches and application. Switzerland: Birkhauser Verlad Base.

[40] Matzke NJ (2013). Probabilistic historical biogeography: new models for founder-event speciation, imperfect detection, and fossils allow improved accuracy and model-testing. Front Biogeogr.

[41] Ree RH, Sanmartín I (2018). Conceptual and statistical problems with the DEC+J model of founder-event speciation and its comparison with DEC via model selection. J Biogeogr.

[42] Ree RH, Moore BR, Webb CO, Donoghue MJ (2005). A likelihood framework for inferring the evolution of geographic range on phylogenetic trees. Evolution.

[43] Ronquist F (1997). Dispersal-vicariance analysis: a new approach to the quantification of historical biogeography. Syst Biol.

[44] Landis MJ, Matzke NJ, Moore BR, Huelsenbeck JP (2013). Bayesian analysis of biogeography when the number of areas is large. Syst Biol.

[45] Zachos J, Pagani M, Sloan L, Thomas E, Billups K (2001). Trends, rhythms, and aberrations in global climate 65 ma to present. Science.

[46] Tiffney BH, Manchester SR (2001). The use of geological and paleontological evidence in evaluating plant phylogeographic hypotheses in the north hemisphere tertiary. J Plant Sci.

[47] Wolfe JA, Leopold EB, Hopkins DM (1967). Neogene and early quaternary vegetation of northwestern North America and northeastern Asia. The Bering band bridge.

[48] Wolfe JA, Graham A (1972). An interpretation of Alaskan tertiary floras. Floristics and paleofloristics of Asia and eastern North America.

[49] Ruddiman WF, Kutzbach JE (1990). Late Cenozoic plateau uplift and climate change. Trans R Soc Edinb Earth Sci.

[50] Axelrod DI (1986). Analysis of some palaeogeographic and palaeoecologic problems of palaeobotany. Palaeobotanist.

[51] Manabe S, Broccoli AJ (1990). Mountains and arid climates of middle latitudes. Science.

[52] Leopold EB, Denton MF (1987). Comparative age of grassland and steppe east and west of the northern Rocky Mountains. Ann Missouri Bot Gard.

[53] Graham A (1999). Late cretaceous and Cenozoic history of north American vegetation: north of Mexico.

[54] Otofuji Y, Matsuda T, Nohda S (1985). Opening mode of the Japan Sea inferred from the palaeomagnetism of the Japan arc. Nature.

[55] Otofuji Y, Itaya T, Matsuda T (1991). Rapid rotation of Southwest Japan—paleomagnetism and K-Ar ages of Miocene volcanic-rocks of Southwest Japan. Geophys J Int.

[56] Baba AK, Matsuda T, Itaya T, Wada Y, Hori N, Yokoyama M, Eto N, Kamei R, Zaman H, Kidane T, Otofuji Y (2007). New age constraints on counter-clockwise rotation of NE Japan. Geophys J Int.

[57] Haq BU, Hardenbol J, Vail PR (1987). Chronology of fluctuating sea levels since the Triassic. Science.

[58] Marincovich Jr L, Gladenkov AY (1999). Evidence for an early opening of the Bering Strait. Nature.

[59] Lagoe MB, Eyles CH, Eyles N, Hale C (1993). Timing of late Cenozoic tidewater glaciation in the far North Pacific. Geol Soc Am Bull.

[60] White JM, Ager TA, Adam DP, Leopold EB, Liu G, Jetté H, Schweger CE (1997). An 18 million year record of vegetation and climate change in northwestern Canada and Alaska: tectonic and global climatic correlates. Palaeogeogr Palaeoclimatol Palaeoecol.

[61] Sigel EM, Windham MD, Haufler CH, Pryer KM (2014). Phylogeny, divergence time estimates, and phylogeography of the diploid species of the *Polypodium vulgare* complex (Polypodiaceae). Syst Bot.

[62] Lagomarsino LP, Condamine FL, Antonelli A, Mulch A, Davis CC (2016). The abiotic and biotic drivers of rapid diversification in Andean bellflowers (Campanulaceae). New Phytol.

[63] Pérez-Escobar OA, Chomicki G, Condamine FL, Karremans AP, Bogarín D, Matzke NJ, Silvestro D, Antonelli A (2017). Recent origin and rapid speciation of Neotropical orchids in the world’s richest plant biodiversity hotspot. New Phytol.

[64] Antonelli A, Nylander JAA, Persson C, Sanmartín I (2009). Tracing the impact of the Andean uplift on Neotropical plant evolution. Proc Natl Acad Sci U S A.

[65] Wei XX, Yang ZY, Li Y, Wang XQ (2010). Molecular phylogeny and biogeography of *Pseudotsuga* (Pinaceae): insights into the floristic relationship between Taiwan and its adjacent areas. Mol Phylogenet Evol.

[66] Cao YN, Comes HP, Sakaguchi S, Chen LY, Qiu XY (2016). Evolution of East Asia’s Arcto-tertiary relict *Euptelea* (Eupteleaceae) shaped by late Neogene vicariance and quaternary climate change. BMC Evol Biol.

[67] Herbert TD, Lawrence KT, Tzanova A, Peterson LC, Caballero-Gill R, Kelly CS (2016). Late Miocene global cooling and the rise of modern ecosystems. Nat Geosci.

[68] Li CX, Lu SG, Ma JY, Gai YH, Yang Q (2014). Phylogeographic history of the woodwardioid ferns, including species from Himalaya. Palaeoworld.

[69] Wang WP, Hwang CY, Lin TP, Hwang SY (2003). Historical biogeography and phylogenetic relationships of the genus *Chamaecyparis* (Cupressaceae) inferred from chloroplast DNA polymorphism. Plant Syst Evol.

[70] Xiang KL, Zhao L, Erst AS, Yu SX, Jabbour F, Wang W (2017). A molecular phylogeny of *Dichocarpum* (Ranunculaceae): implications for eastern Asian biogeography. Mol Phylogenet Evol.

[71] Huang SF (2011). Historical biogeography of the flora of Taiwan. J Taiwan Mus.

