# Supplementary material for: Biogeography of Coptis Salisb. (Ranunculales, Ranunculaceae, Coptidoideae), an Eastern Asian and North American genus
Source: BMC Evol Biol. 2018 May 24;18:74. doi: 10.1186/s12862-018-1195-0 (PMC5968522; doi:10.1186/s12862-018-1195-0)
Supplement: Supplementary file 1 — Table S1. GenBank accession numbers and vouchers/references for the sequences used in this study. Table S2. Manual dispersal multipliers. (PDF 36 kb) [file 12862_2018_1195_MOESM1_ESM.pdf]

Additional file 1

Table S1 GenBank accession numbers and vouchers/references for the sequences used in this study

Table S2 Manual dispersal multipliers

Table S1 GenBank accession numbers and vouchers/references for the sequences used in this study

Taxon	GenBank accession number						Voucher/References
	rbcL	*trnL* intron	*trnL-F* spacer	*trnD-trnT*	*trnH-psbA*	ITS	
Coptis aspleniifolia Salisb.	[1]AB163777	[1]AB163758	[1]AB163738	[1]AB159551	[1]AB163747	[2]KR697579	[1]Y.Terunuma (Unpublished), [2]Jim Bjar 45
C. chinensis Franch.	[1]AB163775	[1]AB163756	[1]AB163736	[1]AB159549	[2]JN862867	[2]JN862853	[1]Y.Terunuma (Unpublished), [2]Cc(JF)02(CMMI)
C. deltoidea C.Y. Cheng & P.G. Xiao	[1]AB163774	[1]AB163755	[1]AB163735	[1]AB159548	[2]JN862873	[2]JN862856	[1]Y.Terunuma (Unpublished), [2]Cd(HL)04(CMMI)
C. japonica var. *anemonifolia* (Siebold & Zucc.) H. Ohba	[1]AB163764	[2]MG991242	[2]KR697588	[1]AB159555	[1]AB163752	[3]AB695574	[1]Y.Terunuma (Unpublished), [2]Tokiko Takenchi 525,[3]J.Shinozaki (Unpublished)
C. laciniata A. Gray	AB163778	AB163759	AB163739	AB159552	AB163748	N/A	Y. Terunuma (Unpublished)
C. lutescens Tamura	[1]AB163766	[1]AB159520	[1]AB159517	[1]AB159518	[1]AB159519	[2]AB695607	[1]Y.Terunuma (Unpublished), [2]J.Shinozaki(Unpublished)
C. morii Hayata	[1]AB163770	[2]MG991243	[2]KR697587	[1]AB159527	[2]KR697584	[2]MG991244	[1]Y.Terunuma (Unpublished), [2]Chen et al.20110736 (PE)
C. occidentalis (Nutt.) Torr. & A. Gray	AB163779	AB163760	AB163740	AB159553	AB163749	N/A	Y. Terunuma (Unpublished)
C. omeiensis (Chen) C.Y. Cheng	[1]AB163776	[1]AB163757	[1]AB163737	[1]AB159550	[2]JN862874	[3]HQ829630	[1]Y.Terunuma(Unpublished), [2]Co(ZF)04(CMMI), [3]HLQC1011-01
C. quinquefolia Miq.	[1]AB163767	[1]AB159524	[1]AB159522	[1]AB159521	[2]KR697585	[2]KR697577	[1]Y.Terunuma(Unpublished) ,[2]G.Mwrota & T. Takahashi 29031
C. quinquesecta W.T. Wang	AB163772	AB159544	AB159546	AB159543	AB159545	[2]KR697580	[1]Y.Terunuma(Unpublished), [2]Sino-Russia Yunnan Exped. 2471
C. ramosa (Makino) Tamura	AB163769	AB159533	AB159535	AB159532	AB159534	N/A	Y. Terunuma (Unpublished)
C. teeta Wall.	[1]AB163773	[1]AB163754	[1]AB163734	[1]AB159547	[2]JN862876	[2]JN862858	[1]Y.Terunuma (Unpublished), [2]Ct(ZZ)07(CMMI)
C. trifolia (L.) Salisb.	[1]AB163771	[1]AB159538	[1]AB159540	[1]AB159536	[1]AB159539	[2]AB695610	[1]Y.Terunuma (Unpublished), [2]J.Shinozaki (Unpublished)
C. trifoliolata (Makino) Makino	AB163768	AB159529	AB159531	AB159528	AB159530	N/A	Y. Terunuma (Unpublished)
Xanthorhiza simplicissima Marshall	[1]L12669	[2]AB163761	[3]EF437111	[2]AB159554	[2]AB163750	[4]KR697583	[1]Y.-L.Qiu et al., [2]Y.Terunuma (Unpublished), [3]Qiu 91030, [4]S.R. Hill 22188

Table S2 Manual dispersal multipliers

Slice 1: 16-3.5 Ma				
A	B	C	D	E
1	0.3	0.3	0.01	1
	1	1	0.7	0.3
		1	0.3	0.3
			1	0.01
				1
Slice 2: 3.5-Present				
A	B	C	D	E
1	0.7	0.7	0.3	1
	1	1	0.7	0.7
		1	0.3	0.7
			1	0.01
				1
