# Supplementary material for: Biogeography of Coptis Salisb. (Ranunculales, Ranunculaceae, Coptidoideae), an Eastern Asian and North American genus
Source: BMC Evol Biol. 2018 May 24;18:74. doi: 10.1186/s12862-018-1195-0 (PMC5968522; doi:10.1186/s12862-018-1195-0)
Supplement: Supplementary file 2 — Figure S1. Raw PDF outputs from biogeographic estimations in BioGeoBEARS. (PDF 358 kb) [file 12862_2018_1195_MOESM2_ESM.pdf]

Additional file 2

Fig. S1. Raw PDF outputs from biogeographical estimations in BioGeoBEARS.

BioGeoBEARS DIVALIKE on Coptis constrained
ancstates: global optim, 3 areas max. d=0.0301; e=0; j=0; LnL=−22.27

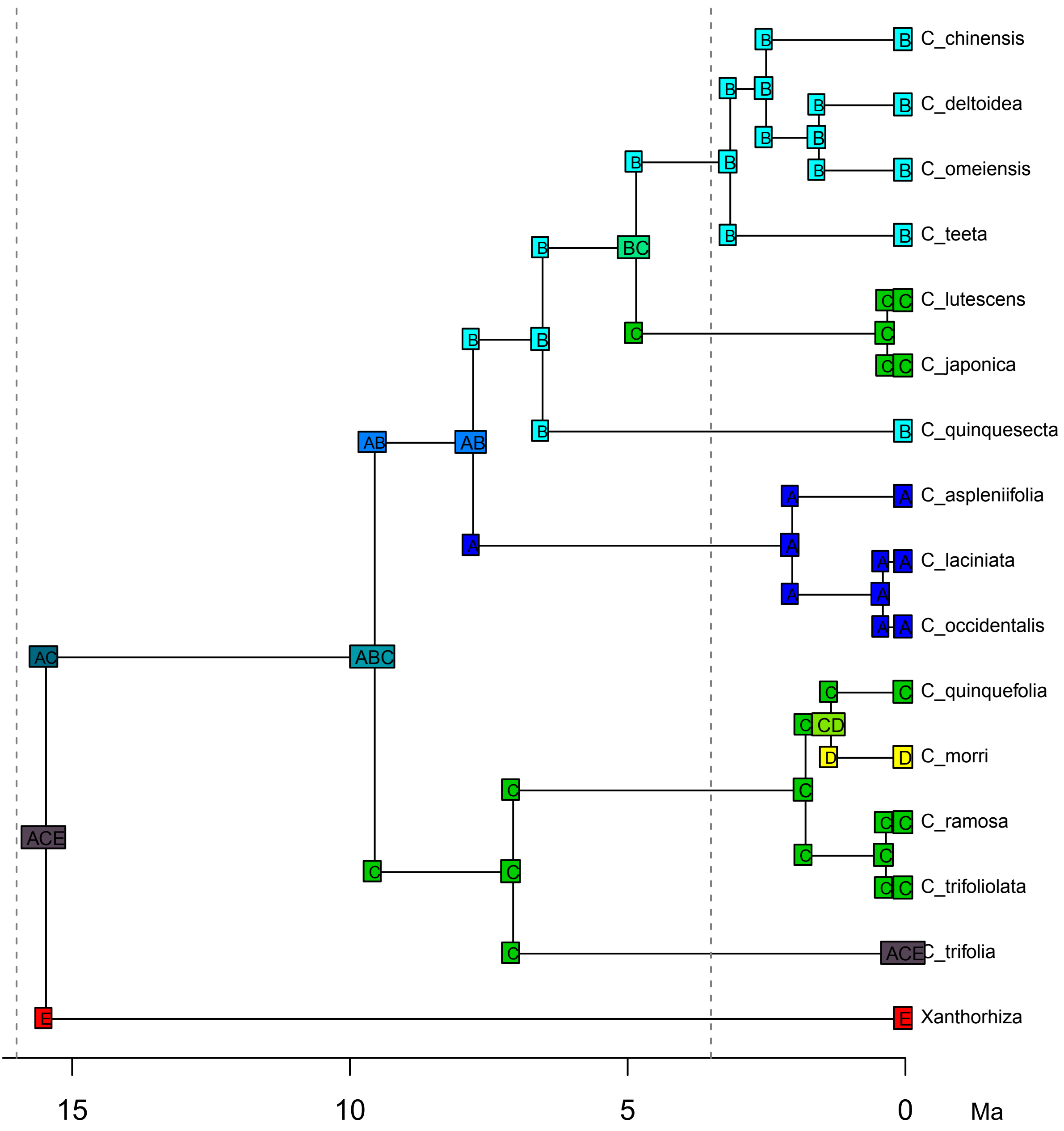

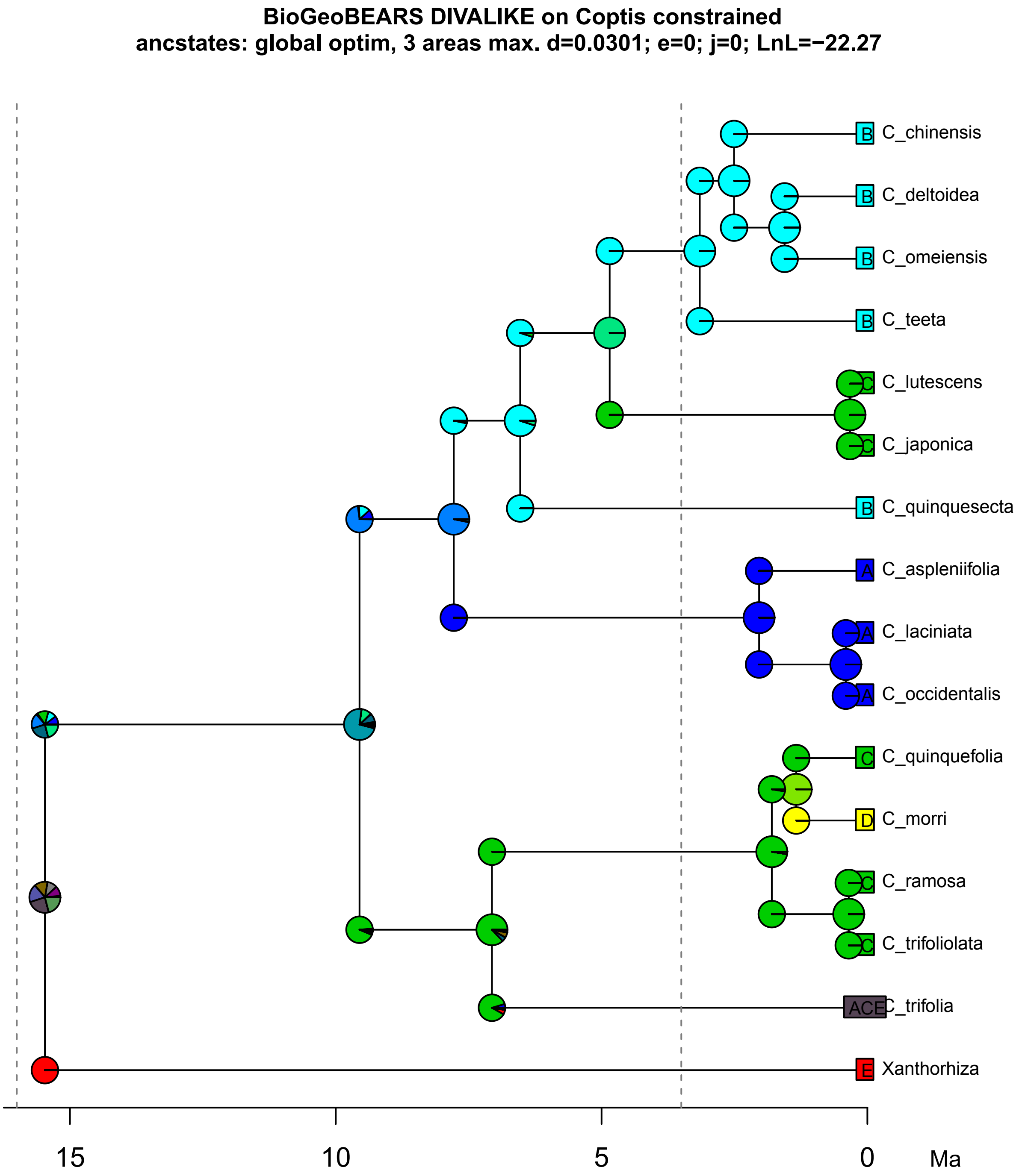

BioGeoBEARS DIVALIKE on Coptis constrained
ancstates: global optim, 3 areas max. d=0.0301; e=0; j=0; LnL=−22.27
B C_chinensis
B C_deltoidea
B C_omeiensis
B C_teeta
C C_lutescens
C C_japonica
B C_quinquesecta
A C_aspleniifolia
A C_laciniata
A C_occidentalis
C C_quinquefolia
D C_morri
C C_ramosa
C C_trifoliolata
ACE C_trifolia
E Xanthorhiza
15
10
5
0
Ma
